# 125. Antimicrobial Stewardship Hospital Activities to Promote Antibiotic Awareness Week 2019, Chicago, IL

**DOI:** 10.1093/ofid/ofab466.327

**Published:** 2021-12-04

**Authors:** Amy P Hanson, Kelly Walblay, Elizabeth Shane, Shannon N Xydis, Massimo Pacilli, Do Young Kim, Stephanie R Black

**Affiliations:** 1 Chicago Department of Public Health, Chicago, Illinois; 2 Rush University Medical Center, Chicago, Illinois

## Abstract

**Background:**

U.S. Antibiotic Awareness Week (USAAW) is an annual campaign to increase knowledge of antimicrobial resistance (AMR) threats and the importance of appropriate antibiotic use. USAAW will be observed November 18-24, 2021 in cadence with World Antimicrobial Awareness Week.

**Methods:**

In October 2019, the Chicago Department of Public Health (CDPH) surveyed 25 Chicago acute care hospital (ACH) antimicrobial stewardship programs (ASPs) via REDCap, an electronic data capture system, to assess their planned activities for USAAW in November 2019. Survey results from 14 (56%) respondent ACHs were collated and disseminated to all 25 ACHs prior to USAAW.

**Results:**

ACH ASP survey responses were categorized by ACH size: smaller hospitals (SH) < 350 beds (n=7) and larger hospitals (LH) > 350 beds (n=7) and displayed in the Table. Nine respondents were Infectious Disease (ID) Pharmacists, 3 were ID Physicians, 1 was an Administrator and 1 was an Infection Prevention Nurse. Among SHs, the ASP was funded for an ID Pharmacist salaried position (FTE) < 0.5 at 4 ACHs, 0.5 in 2, and 1 FTE at 1 ACH. LHs reported ID Pharmacist funding ranging from < 0.5 – 2.5 FTE, with the majority with 1 FTE at 3 LHs. All ACHs reported 0.5 FTE or less ID Physician support for their ASPs. Eleven (79%) of respondent ACHs did not report an annual budget for ASP activities. Ten (71%) ACHs disagreed or strongly disagreed that funding was adequate in the outpatient setting compared to inpatient both for adult and pediatric ASP services. Types of planned activities for USAAW included social media posts, provider education, digital displays, and/or go-live with a new antimicrobial policy. Top three barriers to ASP advancements were financial considerations (n=9), competing responsibilities for ASP leads (n=7), and tied for third was personnel shortages (n=6) and other clinical initiatives with higher priority (n=6).

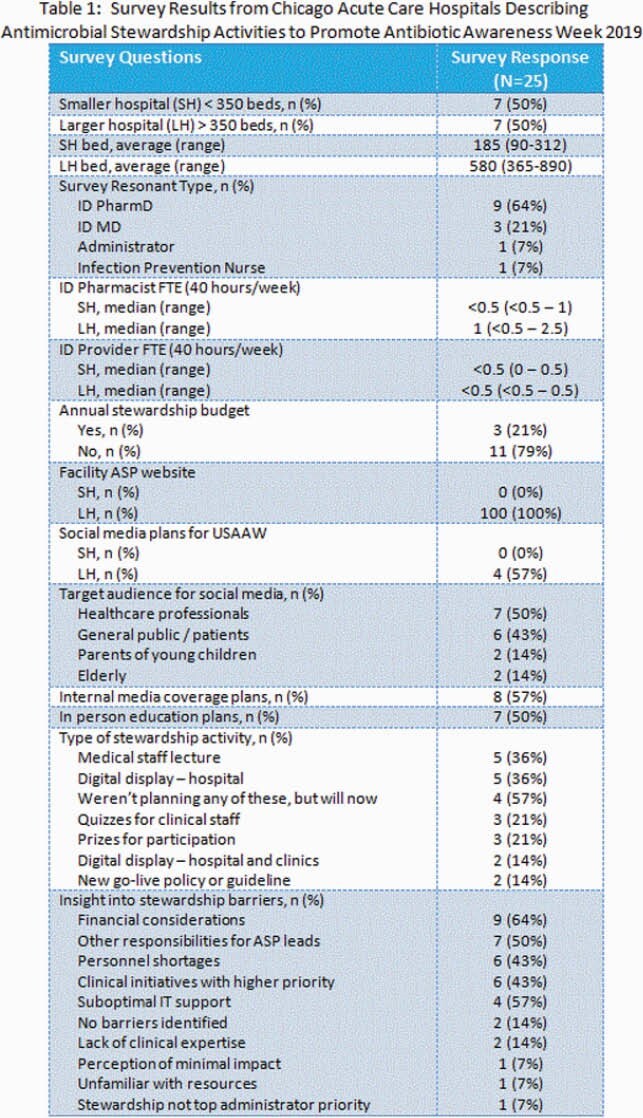

**Conclusion:**

Public Health Departments can facilitate assessment of ACH ASPs within their jurisdiction to identify ways to advance the ASP agenda and combat AMR. A variety of strategies were used by Chicago ACHs to promote ASP initiatives during USAAW. Challenges continue with inadequate funding, especially in outpatient settings.

**Disclosures:**

**All Authors**: No reported disclosures

